# Absence of Telomerase Activity in Malignant Bone Tumors and Soft-Tissue Sarcomas

**DOI:** 10.1080/13577140220127549

**Published:** 2002-03

**Authors:** Nina K. Lauer, Sandra M. Maier, Martin Oberringer, Michael Schulte, Wolf Mutschler, Rainer G. Hanselmann, Manfred Schartl, Stefan J. Scherer, Reiner J. Wirbel

**Affiliations:** 1 Department of Reconstructive Surgery University of Saarland Homburg/Saar D-66421 Germany; 2 Department of Human Genetics University of Saarland Geb. 68 Homburg/Saar D-66421 Germany; 3 Department of Experimental Physics University of Saarland Geb. 22 Saarbrücken 68111 Germany; 4 Department of Trauma Hand and Reconstructive Surgery University of Ulm Ulm 89070 Germany; 5 Department of Surgery Ludwig Maximillian-University of Munich Klinikum Innenstadt Munich 80336 Germany; 6 Department of Physiological Chemistry I, Biocenter University of Wuerzburg Wuerzburg 97074 Germany; 7 Department of Surgery Elisabeth-Hospital Neuwied 56564 Germany; 8 Department of Physiological Chemistry I Biocenter of the University of Wuerzburg Am Hubland Wuerzburg D-97074 Germany

## Abstract

*Purpose:* Telomerase activity appears to play a crucial role in the development of many tumors. More than 80% of all malignant human tumors show an increased telomerase activity. However, conflicting results have been reported about telomerase activity in sarcomas. The aim of the study was to obtain more information about telomerase activity in sarcomas based on a large number of cases.

*Methods:* Telomerase activity was measured in 69 different tumor samples (33 malignant bone tumors and 36 soft tissue sarcomas). Tumor samples were obtained intraoperatively and frozen immediately in liquid nitrogen. Telomerase activity was detected by the telomeric repeat amplification assay (TRAP-assay).

*Results:* Only 7% of the samples showed telomerase activity. No correlation between staging and telomerase activity could be observed.

*Discussion:* The fact that only five out of 69 examined tumor samples showed a telomerase activity provides experimental evidence that in sarcomas the reactivation of telomerase may play a subordinate role. Our results suggest that alternative mechanisms for cell immortalization, yet to be determined, seem to be involved in the development and/or maintenance of soft-tissue sarcomas and malignant bone tumors.


*Sarcoma* (2002) **6**, 43–46

Taylor & Francis healthsciences

**ORIGINAL ARTICLE**

### Absence of telomerase activity in malignant bone tumors and soft-tissue sarcomas

NINA K. LAUER[1], SANDRA M. MAIER[2], MARTIN OBERRINGER[3], MICHAEL SCHULTE[4], WOLF MUTSCHLER[5], RAINER G. HANSELMANN[3], MANFRED SCHARTL[6], STEFAN J. SCHERER[6] & REINER J. WIRBEL[7]

*[1]Department of Reconstructive Surgery, University of Saarland, D-66421 Homburg/Saar, Germany, [2]Department of Human Genetics, University of Saarland, Geb. 68, D-66421 Homburg/Saar, Germany, [3]Department of Experimental Physics, University of Saarland, Geb. 22, 68111 Saarbrücken, Germany, [4]Department of Trauma, Hand and Reconstructive Surgery, University of Ulm, 89070 Ulm, Germany, Department of Experimental Physics, University of Saarland, Geb. 22, 68111 Saarbrücken, Germany, [5]Department of Surgery, Ludwig Maximillian-University of Munich, Klinikum Innenstadt, 80336 Munich, Germany, [6]Department of Physiological Chemistry I, Biocenter, University of Wuerzburg, 97074 Wuerzburg, Germany & [7]Department of Surgery, Elisabeth-Hospital, 56564 Neuwied, Germany*

**Abstract**

*Purpose:* Telomerase activity appears to play a crucial role in the development of many tumors. More than 80% of all malignant human tumors show an increased telomerase activity. However, conflicting results have been reported about telomerase activity in sarcomas. The aim of the study was to obtain more information about telomerase activity in sarcomas based on a large number of cases.

*Methods:* Telomerase activity was measured in 69 different tumor samples (33 malignant bone tumors and 36 soft tissue sarcomas). Tumor samples were obtained intraoperatively and frozen immediately in liquid nitrogen. Telomerase activity was detected by the telomeric repeat amplification assay (TRAP-assay).

*Results:* Only 7% of the samples showed telomerase activity. No correlation between staging and telomerase activity could be observed.

*Discussion:* The fact that only five out of 69 examined tumor samples showed a telomerase activity provides experimental evidence that in sarcomas the reactivation of telomerase may play a subordinate role. Our results suggest that alternative mechanisms for cell immortalization, yet to be determined, seem to be involved in the development and/or maintenance of soft-tissue sarcomas and malignant bone tumors.

**Key words:** *TRAP-assay, cancer, ALT, immortalization*

### Introduction

Telomerase is a ribonucleoprotein that synthesizes G-rich telomeric repeats (TTAGGG) onto chromosomal ends. In this way, telomerase may help to compensate for the gradual shortening of linear molecules replicated by DNA polymerase and contribute to the stability of chromosomes.

It has been determined that telomerase is expressed in 80–90% of human tumors but only rarely in normal adult tissue. It has been also suggested that telomere shortening may be the mechanism underlying the 'mitotic clock' that regulates the limit of normal cell divisions and determines progression into senescence.[1]

Thus, the reactivation of telomerase should enable malignant cells to bypass the normal growth arrest, which is linked to an unlimited proliferation capacity and to immortalization.

The analysis of tumors under the aspect of an increased telomerase activity was carried out with the hope that this enzyme may become an indicator for malignancy and prognosis as well as a target in the therapy of cancer.

Many studies have described increased telomerase activities in carcinoma cell lines as well as malignant tumors of epithelial origin. However, different results exist about telomerase activity in sarcomas.[2–5] Furthermore, these few already existing studies are based on low number of cases.

Correspondence to: Prof. Dr. Manfred Schartl, Department of Physiological Chemistry I, Biocenter of the University of Wuerzburg, Am Hubland, D-97074 Wuerzburg, Germany. Tel.: +49-931-888-4149; Fax: +49-931-888-4150; E-mail: phch1@biozentrum.uni-wuerzburg.de

1357–714X print/1369–1643 online/02/010043–04 © 2002 Taylor & Francis Ltd
DOI: 10.1080/1357714022012754 9

The present study is based on a larger number of cases and different types of tumors. It has been carried out in order to get more information about telomerase activity in sarcomas.

### Material and methods

### *Tissue samples*

The examined tumor samples were obtained from 69 patients (45 males, 24 females) including 17 osteosarcomas, 12 malignant fibrous histiocytomas (MFH), nine leiomyosarcomas, seven Ewing's sarcomas, seven liposarcomas, six chondrosarcomas, three chordomas, three fibrosarcomas, two rhabdomyosarcomas, one clear cell sarcoma, one myxosarcoma and one malignant schwannoma. The tumor, which is listed as a schwannoma, is a malignant peripheral nerve sheath tumor. A further (sub)classification of the rhabdomyosarcomas was not performed. The specimens were taken from vital tumor regions and two independent experienced pathologists confirmed the diagnoses independently; there was no misdiagnosis. Tumor samples were obtained intraoperatively and frozen immediately in liquid nitrogen. Then they were stored at -70°C until further use. Pathologists had examined all tumors and thus the diagnosis was confirmed.

### *TRAP-assay*

Telomerase activity was detected by the telomeric repeat amplification assay (TRAP-assay) using the telomerase detection kit from Boehringer (Mannheim, Germany) based on the method described by Kim *et al.*[6] It allows highly specific amplification of telomerase-mediated elongation products combined with nonradioactive detection following a ELISA protocol.

Small frozen tissue samples were collected and dissolved in lysis buffer. The concentration of protein in the supernatant was determined by using the BCA protein assay kit (Pierce Chemical Co., Rockford, IL, USA). The further procedure followed essentially the instructions of the supplier (Boehringer Mannheim).

As negative control a duplicate of each tissue sample was used which was incubated at 75–80°C for 10 min (prior to the TRAP-assay) to inactivate telomerase. As positive control a telomerase-containing cell extract provided in the kit was used as well as a defined telomerase-positive colon carcinoma. For all tumor specimens, at least two samples were examined which were taken from different parts of the tumor. For the interpretation of the results the mean of the absorbance readings of the negative controls were subtracted from those of the samples. Samples were regarded as telomerase-positive if the difference in absorbance was higher than 0.2 $A_{450nm}$ units. Typical positive values were in a range of 1.367–1.633.

### Results

A total of 69 tumor samples from 69 patients were examined in this study. According to Enneking's classification system of sarcoma[7] most of the tumors were high grade and extracompartmental tumors (stage IIb, $n$=44). Only six of the high-grade tumors were located intracompartmental (stage IIa). Twelve of the 13 low-grade tumors were also extracompartmental tumors (stage Ib).

In six cases there were distant, pulmonal metastases (stage III). Only five of the 69 (7%) showed telomerase activity (see Table 1). In detail these were two Ewing's sarcomas, one leiomyosarcoma, one liposarcoma and one rhabdomyosarcoma (see Table 2).

**Table 1.** *Telomerase activity in malignant bone tumors (MBT) and soft tissue sarcoma (STS)*

| | | | | Stage (Enneking)[2] | | | | | Telomerase activity |
|---|---|---|---|---|---|---|---|---|---|
| | $n^1$ | Tumor specimen | $n^1$ | Ia | Ib | IIa | IIb | III | + |
| MBT | 33 | Osteosarcoma | 17 | | 1 | | 14 | 2 | 0/17 |
| | | Ewing's sarcoma | 7 | | | | 6 | 1 | 2/7 |
| | | Chondrosarcoma | 6 | 1 | 3 | | 1 | 1 | 0/6 |
| | | Chordoma | 3 | | 2 | | | 1 | 0/3 |
| STS | 36 | MFH | 12 | | | 3 | 9 | | 0/12 |
| | | Leiomyosarcoma | 9 | | | 1 | 8 | | 1/9 |
| | | Liposarcoma | 7 | | 5 | 1 | 1 | | 1/7 |
| | | Fibrosarcoma | 3 | | 1 | | 2 | | 0/3 |
| | | Rhabdomyosarcoma | 2 | | | 1 | | 1 | 1/2 |
| | | Clear cell sarcoma | 1 | | | | 1 | | 0/1 |
| | | Myxosarcoma | 1 | | | | 1 | | 0/1 |
| | | Schwannoma | 1 | | | | 1 | | 0/1 |
| | 69 | | 69 | 1 | 12 | 6 | 44 | 6 | 5/69 |

[1]$n$=number of tumor samples.
[2]Enneking's surgical staging (7): I, low grade; II, high grade; III, distant metastasis (low grade or high grade); a, intracompartmental; b, extracompartimental.

**Table 2.** *Tumor samples with measurable telomerase activity*

| | Tumor specimen | n | Stage (Enneking) | | | | |
|---|---|---|---|---|---|---|---|
| | | | Ia | Ib | IIa | IIb | III |
| MBT | Ewing's sarcoma | 2 | | | | 1 | 1 |
| STS | Leiomyosarcoma | 1 | | | | 1 | |
| | Liposarcoma | 1 | | | 1 | | |
| | Rhabdomyosarcoma | 1 | | | | | 1 |

In two tumors, in one of the Ewing's sarcoma and in the rhabdomyosarcoma, there were distant metastases presented at the time of diagnosis. Two tumors, one of the Ewing's sarcomas and the leiomyosarcoma, were of stage IIb, whereas the liposarcoma was a high-grade intracompartmental tumor.

Thus, all positives were found amongst high-grade tumors, whereas no low-grade tumor expressed telomerase activity. These findings could lead to the conclusion that high telomerase activity may be related to the high proliferative state of these tumors. However, this difference was statistically not significant.

### Discussion

During the past decade many studies dealing with telomerase activity in carcinoma have been carried out. These studies have shown that the reactivation of telomerase seems to be a dominating mechanism in the oncogenesis of these tumors. Up to now little is known about sarcoma and telomerase activity. The low number of already existing studies dealing with this question are based on a small number of cases and show contradictory results.[2] For example Aue *et al.*[2] reported telomerase activity in eight of 14 skeletal sarcoma samples, whereby the majority of osteosarcomas showed no telomerase activity. Bryan *et al.*[4] found mainly no telomerase activity in tumor cells derived from sarcoma. Our results are in line with recent findings that only a few soft tissue sarcomas display telomerase activity.[2,8]

In the present study these results have been verified based on a larger number of cases as well as different types of sarcoma. The fact that only five out of 69 examined tumor samples showed telomerase activity confirms the impression that in sarcomas the reactivation of telomerase plays only a subordinate role. Consequently, in the oncogenesis of mesenchymal tumors other mechanisms for immortalization seem to be of decisive importance. Although there is a lot of different tumor types of soft tissue sarcomas and malignant bone tumors, there is a tendency that most of these tumors are telomerase-negative. It is also well known in soft tissue and bone sarcomas that there is a marked heterogeneity throughout the tissue, but all tumors were examined by an experienced pathologist and an independent pathologist confirmed the diagnosis. And overall there was no misdiagnosis.

Some authors have already postulated the existence of one or several such mechanisms. Examination of a series of various immortal cell lines provided evidence that telomere elongation can occur without measurable telomerase activity.[9–13] These mechanisms, which are still basically unknown, were called ALT (*a*lternative *l*engthening of *t*elomeres). Up to now various alternative pathways for telomere maintenance have been discussed, such as an intermittent telomerase activity or telomere–telomere recombination that is known from the yeast *Saccharomyces cerevisiae* (summarized in Ref. 14). A new link between telomere maintenance and mismatch repair was recently published.[15] It was proposed that enhanced telomeric recombination in cells with mismatch-repair defects may contribute to cell immortalization and hence tumorigenesis. However, the molecular mechanisms underlying these phenomena are currently unknown.

In the course of some studies[4–6] it has become obvious that all cell lines derived from sarcomas or immortalized fibroblasts were ALT-positive. This observation is in clear contrast to the situation in epithelial cell lines, which show only rarely ALT. A possible explanation for this difference between epithelial and mesenchymal cells and consequently between carcinoma and sarcoma may be their origin from different germ layers.

Another fact worth mentioning is the observation of some authors that sarcoma often show a shortened telomere length.[3,16] This could be the result of the lack of telomerase activity in the majority of sarcoma. Further studies will have to be carried out in order to trace the exact mechanism and differences of tumor growth in the case of sarcoma in contrast to carcinoma. Such differences might be of vital importance for the development of therapeutical measures.

### Acknowledgements

For excellent technical assistance we thank Christin Weisser and Martina Jennewein. This work was supported by a fellowship of the Interdisziplinäres Zentrum für Klinische Forschung Würzburg to S.J.S.

### References

1. Rhyu MS. Telomeres, telomerase and Immortality. *J Natl Cancer Inst* 1995; 87: 884–94.

2. Aue G, Muradlidhar B, Schwartz HS, Butler MG. Telomerase activity in skeletal sarcomas. *Ann Surg Oncol* 1998; 5: 627–34.
3. Engelhardt M, Albanell J, Drullinsky P, *et al.* Relative contribution of normal and neoplastic cells determines telomerase activity and telomerase length in primary cancers of the prostate, colon, and sarcoma. *Clin Cancer Res* 1997; 3: 1849–57.
4. Bryan TM, Englezou A, Dalla-Pozza L, *et al.* Evidence for an alternative mechanism for maintaining telomere length in human tumors and tumor-derived cell lines. *Nat Med* 1997; 3: 1271–74.
5. Reddel RR, Bryan TM, Murnane JP. Immortalized cells with no detectable telomerase activity. A review. *Biochemistry* 1997; 62: 1254–62.
6. Kim NW, Piatysek MA, Prowse KR, *et al.* Specific association of human telomerase activity with immortal cells and cancer. *Science* 1994; 266: 2011–5.
7. Enneking WF. *Musculoskeletal tumor surgery*. New York, London, Melbourne: Churchill Livingstone, 1983.
8. Yoo J, Robinson RA. Expression of telomerase activity and telomerase RNA in human soft tissue sarcomas. *Arch Pathol Lab Med* 2000; 124: 393–7.
9. Bryan TM, Englezou A, Gupta J, *et al.* Telomere elongation in immortal human cells without detectable telomerase activity. *EMBO J* 1995; 14: 4240–8.
10. Bryan TM, Reddel RR. Telomere dynamics and telomerase activity in *in vitro* immortalised human cells. *Eur J Cancer* 1997; 33: 767–73.
11. Bryan TM, Marusic L, Bacchetti S, *et al.* The telomere lenghtening mechanism in telomerase-negative immortal human cells does not involve the telomerase RNA subunit. *Hum Mol Genet* 1997; 6: 921–6.
12. Wellinger RJ, Ethier K, Labrecque P, Zakian, VA. Evidence for a new step in telomere maintenance. *Cell* 1996; 85: 423–33.
13. Zakian VA. Life and cancer without telomerase. *Cell* 1997; 91: 1–3
14. Biessmann H, Mason JM. Telomere maintenance without telomerase. *Chromosoma* 1997; 106: 63–9.
15. Rizki A, Lundblad V. Defects in mismtach repair promote telomerase-independent proliferation. *Nature* 2001; 411: 713–6.
16. Schneider-Stock R, Epplen C, Radig K, *et al.* On telomere shortening in soft-tissue tumors. *J Cancer Res Clin Oncol* 1998; 124: 165–71.

## References

[PDF_00282] Aue G., Muralidhar B., Schwartz H. S., Butler M. G. (1998). Telomerase activity in skeletal sarcomas.. Ann Surg Oncol.

[PDF_00320] Biessmann H., Mason J. M. (1997). Telomere maintenance without telomerase.. Chromosoma.

[PDF_00290] Bryan T. M., Englezou A., Dalla-Pozza L., Dunham M. A., Reddel R. R. (1997). Evidence for an alternative mechanism for maintaining telomere length in human tumors and tumor-derived cell lines.. Nat Med.

[PDF_00305] Bryan T. M., Englezou A., Gupta J., Bacchetti S., Reddel R. R. (1995). Telomere elongation in immortal human cells without detectable telomerase activity.. EMBO J.

[PDF_00311] Bryan T. M., Marusic L., Bacchetti S., Namba M., Reddel R. R. (1997). The telomere lengthening mechanism in telomerase-negative immortal human cells does not involve the telomerase RNA subunit.. Hum Mol Genet.

[PDF_00308] Bryan T. M., Reddel R. R. (1997). Telomere dynamics and telomerase activity in in vitro immortalised human cells.. Eur J Cancer.

[PDF_00285] Engelhardt M., Albanell J., Drullinsky P., Han W., Guillem J., Scher H. I., Reuter V., Moore M. A. (1997). Relative contribution of normal and neoplastic cells determines telomerase activity and telomere length in primary cancers of the prostate, colon, and sarcoma.. Clin Cancer Res.

[PDF_00297] Kim N. W., Piatyszek M. A., Prowse K. R., Harley C. B., West M. D., Ho P. L., Coviello G. M., Wright W. E., Weinrich S. L., Shay J. W. (1994). Specific association of human telomerase activity with immortal cells and cancer.. Science.

[PDF_00294] Reddel R. R., Bryan T. M., Murnane J. P. (1997). Immortalized cells with no detectable telomerase activity. A review.. Biochemistry (Mosc).

[PDF_00272] Rhyu M. S. (1995). Telomeres, telomerase, and immortality.. J Natl Cancer Inst.

[PDF_00322] Rizki A., Lundblad V. (2001). Defects in mismatch repair promote telomerase-independent proliferation.. Nature.

[PDF_00325] Schneider-Stock R., Epplen C., Radig K., Oda Y., Dralle H., Hoang-Vu C., Epplen J. T., Roessner A. (1998). On telomere shortening in soft-tissue tumors.. J Cancer Res Clin Oncol.

[PDF_00315] Wellinger R. J., Ethier K., Labrecque P., Zakian V. A. (1996). Evidence for a new step in telomere maintenance.. Cell.

[PDF_00302] Yoo J., Robinson R. A. (2000). Expression of telomerase activity and telomerase RNA in human soft tissue sarcomas.. Arch Pathol Lab Med.

[PDF_00318] Zakian V. A. (1997). Life and cancer without telomerase.. Cell.

